# Clinical Impact of the BIOFIRE Blood Culture Identification 2 Panel in Adult Patients with Bloodstream Infection: A Multicentre Observational Study in the United Arab Emirates

**DOI:** 10.3390/diagnostics13142433

**Published:** 2023-07-21

**Authors:** Abiola Senok, Laila Al Dabal, Mubarak Alfaresi, Maya Habous, Handan Celiloglu, Safia Bashiri, Naama Almaazmi, Hassan Ahmed, Ayman A. Mohmed, Omar Bahaaldin, Maimona Ahmed Elsiddig Elimam, Irfan Hussain Rizvi, Victory Olowoyeye, Michaela Powell, Basel Salama

**Affiliations:** 1 College of Medicine, Mohammed Bin Rashid University of Medicine and Health Sciences, Dubai P.O. Box 505055, United Arab Emirates; victory.olowoyeye@students.mbru.ac.ae; 2 Infectious Diseases Unit, Rashid Hospital, Dubai P.O. Box 4545, United Arab Emirates; lmdabal@dha.gov.ae (L.A.D.); sbashiri@dha.gov.ae (S.B.); naalmaazmi@dha.gov.ae (N.A.); haaahmed@dha.gov.ae (H.A.); 3 Pathology and Laboratory Medicine, Zayed Military Hospital, Abu Dhabi P.O. Box 72763, United Arab Emirates; mubarak.alfarsi@msc.mil.ae; 4 Microbiology and Infection Control Unit, Pathology Department, Rashid Hospital, Dubai P.O. Box 4545, United Arab Emirates; mmhabou@dha.gov.ae (M.H.); obnuman@dha.gov.ae (O.B.); maelimam@dha.gov.ae (M.A.E.E.); 5 Microbiology Department, Mediclinic City Hospital, Dubai Healthcare City, Dubai P.O. Box 505004, United Arab Emirates; handanyol@hotmail.com (H.C.); irfan.hussein@mediclinic.ae (I.H.R.); 6 Intensive Care Unit, Sheikh Khalifa General Hospital, Umm Al Quwain P.O. Box 499, United Arab Emirates; ayman.ali@skgh.ae; 7 Data Science Department, bioMérieux Inc., Salt Lake City, UT 84108, USA; michaela.powell@biomerieux.com; 8 Medical Affairs, bioMérieux, Dubai P.O. Box 505201, United Arab Emirates; basel.salama@biomerieux.com

**Keywords:** BIOFIRE Blood Culture Identification Panel, BCID, BCID2, bloodstream infection, sepsis, blood culture, targeted antimicrobial therapy, optimal antimicrobial therapy, rapid molecular diagnostics, automated multiplex PCR

## Abstract

Rapid pathogen identification is key to the proper management of patients with bloodstream infections (BSIs), especially in the intensive care setting. This multicentre study compared the time to pathogen identification results in 185 patients admitted to intensive care with a confirmed BSI, using conventional methods (*n* = 99 patients) and upon implementation of the BIOFIRE^®^ Blood Culture Identification 2 (BCID2) Panel, a rapid molecular test allowing for the simultaneous identification of 43 BSI-related nucleic acids targets (*n* = 86 patients). The median time to result informing optimal antibiotic therapy was significantly shorter following the implementation of the BCID2 Panel (92 vs. 28 h pre vs. post BCID2 implementation; *p* < 0.0001). BCID2 usage in addition to conventional methods led to the identification of at least one pathogen in 98.8% patients vs. 87.9% using conventional methods alone (*p* = 0.003) and was associated with a lower 30-day mortality (17.3% vs. 31.6%, respectively; *p* = 0.019). This study at three intensive care units in the United Arab Emirates therefore demonstrates that, in addition to conventional microbiological methods and an effective antimicrobial stewardship program, the BCID2 Panel could improve the clinical outcome of patients admitted to the intensive care unit with a confirmed BSI.

## 1. Introduction

Bloodstream infections are associated with high morbidity and mortality, especially in the intensive care setting [1,2,3,4,5,6]. Current guidelines for empirical treatment of bloodstream infections call for the use of broad-spectrum antibiotics and a prompt antimicrobial adjustment upon pathogen identification, in accordance with the local antimicrobial stewardship guidelines [7,8]. Rapid detection of causative pathogens and of potential resistance determinants in blood cultures is crucial for a timely administration of optimal therapy and for improving patients’ survival [3,9,10,11,12]. Indeed, delayed administration of adequate antimicrobial therapy, due to either too long time to pathogen identification or antimicrobial resistance, increases mortality [1,2,3,4,5,6,9,10,11,12].

In recent years, approaches to shorten the time to identification of pathogens have been developed. Among them, rapid molecular diagnostics based on multiplex PCR allow for the identification of a broad panel of pathogens as well as of antibiotics resistance genes directly from positive blood cultures [3,9,10]. The BIOFIRE^®^ Blood Culture Identification 2 (BCID2) Panel (bioMérieux, Marcy l’Etoile, France) is a well-validated assay that allows for the simultaneous identification of 43 nucleic acid targets associated with bloodstream infections (26 bacterial genera/species, 7 fungal species, and 10 resistance markers) within about 1 h. It is FDA-approved, CE-marked, and locally approved in the United Arab Emirates (UAE). The BCID2 Panel (and prior to that, its first-generation BCID) demonstrated high diagnostic accuracy compared to conventional microbiological methods [13,14,15,16,17,18,19,20,21,22,23,24,25]. Its implementation is associated with a significant decrease in time to result (pathogen and resistance gene identification) or time to optimal antimicrobial therapy, compared to conventional methods [14,16,19,22,25,26,27,28]. Its use led to treatment adjustments in 22–45% patients in several reported studies [19,22,25,27,28].

Current guidelines recommend the addition of rapid diagnostic testing to an effective antimicrobial stewardship program (ASP) [7,8]. A randomised study indeed demonstrated that combining the BCID panel with antimicrobial stewardship provided greater clinical benefit compared to BCID alone or to conventional methods [27].

To the best of our knowledge, no studies so far have evaluated the clinical impact of BCID2 usage in an intensive care unit (ICU) setting in the UAE. This multicentre study aimed at evaluating, for the first time in the UAE, the clinical impact of the BCID2 Panel on the time to result informing targeted antimicrobial therapy in adult ICU patients with a bloodstream infection. We compared results of patients recruited prospectively for a period of 6 months upon implementation of the BCID2 Panel (performed in addition to conventional microbiological methods) to retrospective data of patients evaluated using conventional methods alone, during a 6-month period preceding the implementation of BCID2. We hypothesised that in patients with a bloodstream infection, the BCID2 Panel will have a higher diagnostic yield and a shorter time to results on microbial and resistance gene identification compared to conventional methods. An earlier targeted antimicrobial therapy is expected to reduce the exposure to broad-spectrum antibiotics and the rate of inappropriate antibiotic treatment, thereby potentially improving patients’ outcomes.

We showed that the implementation of the BCID2 Panel was associated with a significantly shorter median time to result informing optimal (i.e., targeted) therapy and a reduced 30-day mortality in ICU patients with a bloodstream infection in our UAE hospital setting.

## 2. Materials and Methods

### 2.1. Study Design and Participants

A multicentre observational cohort study was conducted in adult patients (≥18 and ≤85 years-old) admitted in the ICU with a confirmed bloodstream infection (i.e., a positive blood culture) to evaluate the impact of the BIOFIRE^®^ Blood Culture Identification 2 (BCID2) Panel (bioMérieux, Marcy l’Etoile, France) on the time to result informing optimal (i.e., targeted) antimicrobial therapy. Targeted antimicrobial therapy was defined as any regimen that is active against the causative pathogen that ultimately grew in culture and to which the organism was susceptible. The time to result informing targeted therapy was defined as the time duration from collection of the first positive blood culture sample to obtention of the test result (i.e., pathogen identification per conventional methods or the BCID2 Panel) [29,30,31]. The time to result instructing targeted antimicrobial therapy for any patient who did not fall within evidence-based guidelines was adjudicated by the infectious disease physicians on the study team. Two study groups were compared. The first study group included retrospective data from ICU patients with a confirmed bloodstream infection during the six months immediately preceding the implementation of the BCID2 Panel at three participating centres (Dubai, United Arab Emirates): Rashid Hospital (June to November 2020), Mediclinic City Hospital (August 2020 to January 2021), and Sheikh Khalifa General Hospital (June to November 2019). In this study group (thereafter referred to as pre-BCID2), pathogen identification was conducted according to conventional microbiological methods only. The second study group included prospective data of ICU patients with a confirmed bloodstream infection, collected for a period of six months (February to July 2022) upon implementation of the BCID2 Panel at the same three participating centres. Pathogen identification in this study group (thereafter referred to as BCID2) was based on the results of the BCID2 Panel, in addition to standard conventional methods. Besides the time to pathogen identification results, other clinical outcomes were compared between pre-BCID2 and BCID2 patients, including the 30-day mortality, the duration of empirical antibiotics use, the number of prescribed empirical antibiotics, and the percentage of patients with antibiotics de-escalation (defined as the switch from empirical to targeted antibiotics following pathogen identification results). The duration of ICU stay could not be calculated, as the date of discharge from the ICU was not documented in this study. Patients’ demographics, comorbidities and vital signs were recorded at ICU admission. In the absence of a standardised scoring system among the three participating centres [32], disease severity and organ dysfunction at ICU admission were assessed based on documented vital signs.

The study was conducted in accordance with the Declaration of Helsinki and approved by the Dubai Scientific Research Ethics Committee (DSREC), Dubai Health Authority (approval number DSREC-10/2021_06, dated 2 November 2021) and the Mediclinic Middle East (MCME) Research and Ethics Committee (approval number MCME.CR.225.MCIT.2021, dated 30 November 2021). An informed consent was waived by the DSREC Dubai Health Authority and the MCME Research and Ethics Committee because blood samples were collected and blood cultures performed as part of routine clinical care. No blood samples were drawn specifically for this study, the study did not involve patient contact, and patient data were de-identified.

### 2.2. Sample Collection and Processing

Blood samples were collected solely as part of standard clinical care for ICU patients. Blood cultures were performed immediately after blood collection as per the standard protocol in place at the respective participating centres, using automated blood culture systems. The first positive blood culture data were included in the study analysis.

For the retrospective data, conventional analysis of positive blood cultures involved the performance of Gram stain, bacterial identification, and antimicrobial susceptibility testing using an automated platform, as described in Section 2.3.1. For the prospective data, positive blood cultures were further analysed using the BCID2 Panel within one hour of detected growth by the automated blood culture system, as described in Section 2.3.2. Simultaneously to the BCID2 assay, Gram stain and routine bacterial identification and antimicrobial susceptibility testing using an automated platform were conducted, as per the standard protocol at each participating centre (see Section 2.3.1). In case of discrepancy between the results from the BCID2 Panel and the standard conventional methods, the latter was used as the gold standard for patient management.

The pathogen identification results (conventional methods or BCID2, as applicable) were communicated within one hour of obtention to the ICU physician and the antibiotic stewardship program (ASP) team, including the study’s infectious disease physician. The ASP team reviewed the results and communicated recommendations on an antimicrobial regimen to the ICU team for implementation, in line with current ASP guidelines at the respective hospitals.

### 2.3. Laboratory Methods

#### 2.3.1. Automated Blood Culture and Conventional Microbiological Testing

Blood culture and conventional microbiological testing at the three participating centres were done according to the international recommended protocols and the Clinical and Laboratory Standards Institute (CLSI) guidelines [33,34,35], as follows.

At Rashid Hospital, Gram stain was done manually (SURECHEM, Suffolk, UK). The recovery and detection of microorganisms were conducted using the automated BACT/ALERT^®^ VIRTUO^®^ Microbial Detection System (bioMérieux) with the resin-based aerobic (BACT/ALERT^®^ FA Plus), anaerobic (BACT/ALERT^®^ FN Plus) and aerobic and facultative anaerobic (BACT/ALERT^®^ PF Plus) bottles (bioMérieux), or the BD BACTEC™ Automated Blood Culture System (Becton Dickinson, Franklin Lakes, NJ, USA) with the resin-based BD BACTEC™ Plus Aerobic and Anaerobic media. Positively flagged blood culture bottles were sub-cultured on blood-agar plates, and isolated colonies were identified by matrix-assisted laser desorption ionisation time-of-flight (MALDI-TOF) using the VITEK MS^®^ automated mass spectrometry microbial identification system (bioMérieux). 

At Mediclinic City Hospital, Gram stain was done manually (Dagatron Auto Stainer AT-3002, Biomed Global, Gyeonggi-do, Republic of Korea). The recovery and detection of microorganisms were conducted using the automated BACT/ALERT^®^ VIRTUO^®^ Microbial Detection System (bioMérieux) with the BACT/ALERT^®^ FA Plus, BACT/ALERT^®^ FN Plus, and BACT/ALERT^®^ PF Plus bottles (bioMérieux). Positively flagged blood culture bottles were sub-cultured on blood-agar plates, and isolated colonies were identified using the colorimetric VITEK 2*^®^* Compact automated system (bioMérieux).

At Sheikh Khalifa General Hospital, Gram stain was done using the RAL Stainer automated staining unit (bioMérieux). The recovery and detection of microorganisms were conducted using the BD BACTEC™ Automated Blood Culture System (Becton Dickinson, Franklin Lakes, NJ, USA) with the resin-based BD BACTEC™ Plus Aerobic and Anaerobic media. Positively flagged blood culture bottles were sub-cultured on blood-agar plates, and isolated colonies were identified using the colorimetric VITEK 2*^®^* Compact automated system (bioMérieux).

For antimicrobial susceptibility testing (AST) at the three centres, 0.5 McFarland suspensions were prepared following the manufacturer’s protocol (DensiCHEK Plus, bioMérieux) and inoculated into specific VITEK 2^®^ susceptibility cards (bioMérieux), except for colistin sensitivity testing at Rashid Hospital and Sheikh Khalifa General Hospital, which was performed by broth microdilution (ComASP™ Colistin, Liofilchem, Italy). Interpretation of resistance phenotypes was performed using the Advanced Expert System^®^ (AES) software (version 2.0.0; bioMérieux). Interpretation of susceptibility profiles was done using the VITEK 2^®^ software version 9.02.

#### 2.3.2. BIOFIRE Blood Culture Identification 2 (BCID2) Panel

The BIOFIRE^®^ Blood Culture Identification 2 (BCID2) Panel (bioMérieux, Marcy l’Etoile, France) was used in accordance with the manufacturer’s instructions. The BCID2 Panel is a multiplexed nucleic acid test intended for the simultaneous qualitative detection and identification of 43 targets associated with bloodstream infections, including 15 Gram-negative bacteria (*Acinetobacter calcoaceticus-baumannii* complex, *Bacteroides fragilis*, *Haemophilus influenzae*, *Neisseria meningitidis*, *Pseudomonas aeruginosa*, *Stenotrophomonas maltophilia*, *Enterobacterales*, *Enterobacter cloacae* complex, *Escherichia coli*, *Klebsiella aerogenes*, *Klebsiella oxytoca*, *Klebsiella pneumoniae* group, *Proteus* spp., *Salmonella* spp., *Serratia marcescens*), 11 Gram-positive bacteria (*Enterococcus faecalis*, *Enterococcus faecium*, *Listeria monocytogenes*, *Staphylococcus* spp., *Staphylococcus aureus*, *Staphylococcus epidermidis*, *Staphylococcus lugdunensis*, *Streptococcus* spp., *Streptococcus agalactiae*, *Streptococcus pneumoniae*, *Streptococcus pyogenes*), 7 yeast species (*Candida albicans*, *Candida auris*, *Candida glabrata*, *Candida krusei*, *Candida parapsilosis*, *Candida tropicalis*, *Cryptococcus neoformans/gattii*) and 10 antimicrobial resistance genes (carbapenemase genes: IMP, KPC, OXA-48-like, NDM, VIM; colistin resistance gene: *mcr-1*; extended-spectrum beta-lactamase [ESBL] gene: CTX-M; methicillin resistance genes: *mec*A/C, *mec*A/C and MREJ [Methicillin-resistant Staphylococcus aureus (MRSA)]; vancomycin resistance gene: *van*A/B). The BCID2 Panel test was performed on blood culture samples identified as positive by a continuous monitoring blood culture system. Results were available within about one hour from positive blood culture. Results were interpreted in conjunction with Gram stain results, as per the manufacturer’s recommendation.

### 2.4. Statistical Analysis

Statistical analyses were performed using R version 4.1.2. *p*-values < 0.05 were considered statistically significant. For categorical variables, comparison of pre-BCID2 and BCID2 data was performed using Fisher’s exact test. When categorical variables had more than two categories, Fisher’s exact test was implemented using the Monte Carlo simulation. For continuous variables, comparison of pre-BCID2 and BCID2 data was performed using the Wilcoxon rank-sum test. Comparison of BCID2 and pre-BCID2 implementation periods for the primary and secondary aims was performed using one-sided statistical tests.

The time to test result informing optimal treatment was calculated as the time interval between the collection of the first positive blood culture sample and the availability of the test result (susceptibility result in the pre-BCID2 implementation period and BCID2 result or susceptibility result if earlier in the BCID2 implementation period) using the following formula:

Time to test result informing optimal treatment (in hours) = Time of first test result (susceptibility or BCID2) − Time of collection of first positive blood culture sample.

The difference in the mean times to test result informing optimal treatment between the pre-BCID2 and BCID2 implementation periods was evaluated after accounting for centre-to-centre variability using the following linear mixed model:

*t_ij_* = *β*_0_ + *β*_1_
*x_ij_* + *u_j_*

where *t_ij_* is the time to test result informing optimal treatment for patient *i* at centre *j*, *x_ij_* equals either 0 if patient *i* at centre *j* is in the pre-BCID2 implementation period or 1 if patient *i* at centre *j* is in the BCID2 implementation period, and *u_j_* is the random intercept for centre *j*. A *p*-value < 0.05 for $\beta_{1}$ was considered indicative of a statistically significant difference in the mean times to test results informing optimal treatment between the BCID2 implementation periods. The difference in the mean times to results informing optimal treatment between the pre-BCID2 and BCID2 implementation periods, after accounting for centre-to-centre variability, was estimated with the likelihood profile confidence interval for $\beta_{1}$.

The duration of empirical antibiotics use was calculated as the time interval between initiation of the first empirical antibiotics treatment and antibiotics change to targeted therapy (de-escalation) following the pathogen identification result (using conventional methods or BCID2) or the full susceptibility result (whatever was earlier), using the following formula:

Duration of empirical antibiotics use (in days) = Time the antibiotics change was ordered − Time the first empirical antibiotics was initiated.

The false discovery rate of the BCID2 Panel relative to conventional methods, set as the gold standard, was calculated for the BCID2 cohort. It was defined as the ratio of false positive detections by BCID2 (discordant or detected only by BCID2) to the total detections (sum of false positive detections and true positive—i.e., concordant—detections) by BCID2. This calculation only considered pathogens that were detectable by both approaches and thus excluded pathogens that are not tested by the BCID2 Panel (off-panel pathogens).

## 3. Results

### 3.1. Patients’ Characteristics

**Figure 1:**
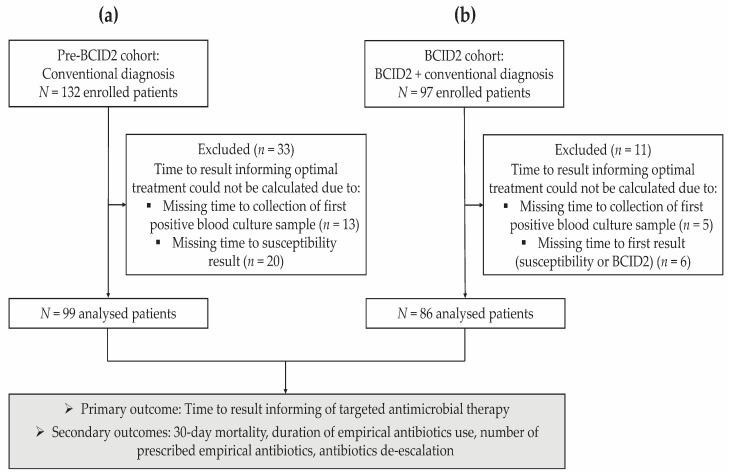
Study flow diagram. (**a**) Pre-BCID2 implementation period. Bloodstream infection diagnosis was based on the results of conventional microbiological methods. The pre-BCID2 cohort included retrospective data from ICU patients with a confirmed bloodstream infection, collected during the immediate six months preceding the implementation of the BCID2 Panel. (**b**) BCID2 implementation period. The BCID2 cohort included prospective data of ICU patients with a confirmed bloodstream infection, collected for a period of six months upon implementation of the BCID2 Panel. Pathogen identification was conducted using the BCID2 Panel, in addition to standard conventional methods. Bloodstream infection diagnosis was based on BCID2 results, unless they were discordant with those of the conventional approach, in which case conventional results were considered for diagnosis.

A total of 229 ICU patients with a confirmed bloodstream infection (BSI), i.e., with a positive blood culture, were enrolled in the study, including 132 patients whose BSI was tested using conventional microbiological methods, pre-implementation of the BCID2 Panel (defining the pre-BCID2 cohort), and 97 patients recruited prospectively and tested using the BCID2 Panel in addition to conventional methods (BCID2 cohort; Figure 1). Of these enrolled patients, 99 pre-BCID2 and 86 BCID2 patients with a calculated time to pathogen identification result (informing optimal targeted therapy) were included in the analysis (Figure 1).

Patients’ characteristics at the time of ICU admission are described in Table 1. Patients were mainly male (70.7% and 65.1% in pre-BCID2 and BCID2 cohorts, respectively). The median (interquartile range [IQR]) ages in the pre-BCID2 and BCID2 study groups were 56 (40–65) and 65 (45–79) years, respectively. Most patients (66.7% pre-BCID2, 76.7% BCID2) presented up to five comorbidities, the commonest comorbidities in both groups being hypertension, diabetes, and cardiac disease. Patients of the BCID2 cohort also had a higher frequency of chronic lung disease compared to patients of the pre-BCID2 cohort (Table 1).

### 3.2. Detected Pathogens’ Characteristics

In the pre-BCID2 implementation phase, pathogens were detected in 87/99 (87.9%) patients by conventional methods, compared to 85/86 (98.8%) patients in the BCID2 implementation phase (using the BCID2 Panel in addition to conventional methods) (Table 2). Only one (1.2%) BCID2 patient vs. 12 (12.1%) pre-BCID2 patients had no detected pathogens (*p* = 0.003). The proportion of patients with single detections was comparable in both study groups (83/99 [83.8%] pre-BCID2 vs. 72/86 [83.7%] BCID2; *p* = 1.000). However, there were significantly more patients with the simultaneous detection of two pathogens during the BCID2 implementation phase compared to pre-BCID2 (13/86 [15.1%] vs. 4/99 [4.0%]; *p* = 0.011; Table 2).

A total of 91 pathogens (in 87 pre-BCID2 patients) and 98 pathogens (in 85 BCID2 patients) were identified (Table S1). The most predominant microorganisms detected by both approaches were *Klebsiella pneumoniae*, *Escherichia coli*, and *Staphylococcus epidermidis. Candida auris* was also frequently detected during the BCID2 implementation period, while it was never detected by conventional methods during the pre-BCID2 implementation period (Table S1).

Identified resistance phenotypes (by conventional methods) and resistance genes (by the BCID2 Panel) are shown in Table S1. Conventional methods identified antimicrobial resistance phenotypes in 11/91 (12.1%) and 4/98 (4.1%) detected pathogens in the pre-BCID2 and BCID2 implementation periods, respectively (Table S1). In comparison, the BCID2 Panel identified 29 resistance genes (29/98 [29.6%]). Resistance genes for Gram-negative bacteria (including *Klebsiella pneumoniae*, *Escherichia coli* and *Klebsiella aerogenes*) were CTX-M (*n* = 13), OXA-48-like (*n* = 3), NDM (*n* = 2), and *mcr-1* (*n* = 1) (Table S1). Two identified *Klebsiella pneumoniae* were simultaneously positive for two resistance genes (CTX-M + NDM, CTX-M + OXA-48-like; Table S1). The *mecA* gene was identified in all detected Gram-positive *Staphylococcus epidermidis* (*n* = 10), and none of the isolates were positive for the *vanA/B* genes (Table S1).

Resistance and susceptibility of isolates to antimicrobial agents in both study phases are shown in Table S2 (pre-BCID2 phase) and Table S3 (BCID2 phase).

We evaluated the concordance in pathogen identification by conventional methods and the BCID2 Panel during the BCID2 implementation period. Of the 98 organisms identified (BCID2 phase; Table S1), most (74/98 [75.5%]) were fully concordant between the two identification methods. These included 70 patients with one identified pathogen and two patients with two co-detected organisms (*Staphylococcus epidermidis* and *Candida auris* in both cases). Of the 24/98 (24.5%) discordant pathogens, four (in two patients) were partially discordant (several species of the genus *Staphylococcus*), 14 (in 13 patients) were identified either by BCID2 (*n* = 5) or conventional culture (*n* = 9), and six (in three of 85 [3.5%] patients) were truly discordant (Table S4). The latter corresponded to two patients with *Elizabethkingia meningoseptica* identified by conventional culture and positive in the BCID2 Panel for *Escherichia coli* (one patient) and for *Candida auris* (second patient) and to one patient with *Streptococcus viridans* identified by conventional culture and positive for *Staphylococcus* spp. in the BCID2 Panel (Table S4). Of the nine pathogens not identified by the BCID2 Panel, four were off-panel microorganisms (*Corynebacterium* spp., *Kytococcus* spp., *Aeromonas sóbria*, *Enterococcus Avium Group D*) and five were on-panel microorganisms and thus corresponded to detection failures (Table S4).

The false discovery rate of BCID2 relative to conventional methods, focusing on pathogens detectable by both approaches (i.e., excluding off-panel pathogens), was calculated based on these concordance results. The false discovery rate, defined as the ratio of false positive detections by BCID2 to the total detections (false positive and true positive detections) by BCID2, was 15/89 or 16.9%.

### 3.3. Time to Result Informing Targeted Therapy in the Pre-BCID2 vs. BCID2 Cohorts

**Figure 2:**
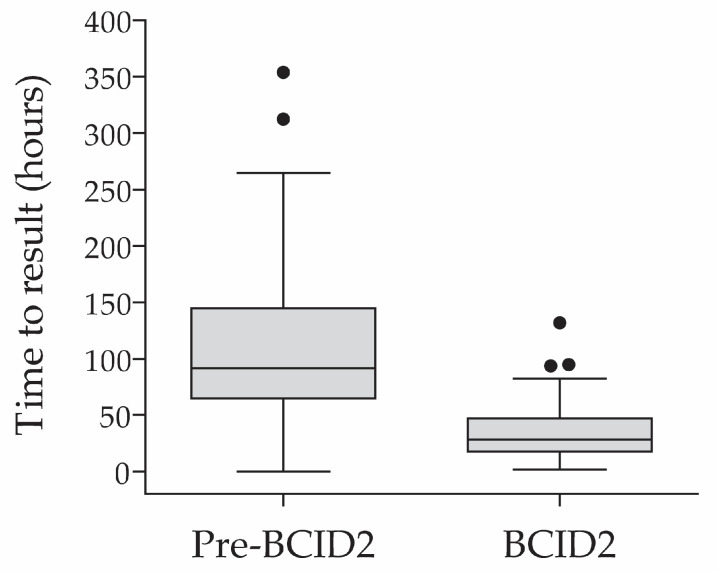
Time to result informing optimal (targeted) therapy before and after implementation of BCID2 testing.

The time to result informing optimal therapy was calculated as the difference between the time of collection of the first positive culture sample and the time to availability of the test result (susceptibility result in the pre-BCID2 study group and BCID2 Panel result or susceptibility result in the BCID2 study group). The median (IQR) times to result informing targeted therapy in the pre-BCID2 and BCID2 phases were 91.7 (64.7–144.9) and 28.1 (17.6–47.1) hours, respectively (*p* < 0.0001; Figure 2).

After accounting for centre-to-centre variability, the estimated difference (95% confidence interval [CI]) of the mean time to result informing optimal treatment between the pre-BCID2 and BCID2 implementation periods was 73.3 (59.8–88.6) hours (linear mixed models, *p* < 0.0001).

### 3.4. Secondary Clinical Outcomes

We compared the 30-day mortality (relative to the day blood culture specimens were drawn) in both study groups. In the pre-BCID2 and the BCID2 study periods, one and five patients, respectively, lacking a documented mortality status were excluded from the comparison. The 30-day mortality rate in the BCID2 study group was significantly lower than that of pre-BCID2 (14/81 [17.3%] vs. 31/98 [31.6%], respectively; *p* = 0.019).

To evaluate the possible impact of BCID2 implementation on antibiotics treatment adjustment, the number and duration of empirical antibiotics usage were compared in both study groups. The number of empirical antibiotics ordered in both study groups (from none to three per patient) was comparable (*p* = 0.394; Table 3).

The duration of empirical antibiotics usage was able to be calculated in 75 pre-BCID2 and 64 BCID2 patients. The median (IQR) duration of treatment with empirical antibiotics was shorter in BCID2 patients (2 [1,2,3,4] days) than in pre-BCID2 patients (3 [1,2,3,4] days), albeit the difference was not statistically significant (*p* = 0.126).

Antibiotics de-escalation, defined as the switch from empirical antibiotics to targeted therapy following test result (pathogen identification or susceptibility result), was comparable in both groups (*p* = 1.000; Table 4).

## 4. Discussion

This multicentre study is, to the best of our knowledge, the first to evaluate the potential clinical benefit of implementing the BCID2 Panel for the management of ICU patients with a confirmed BSI in the United Arab Emirates.

After accounting for centre-to-centre variability, the mean time to results gained using the BCID2 Panel (in addition to conventional methods) compared to using conventional methods alone was 73.3 h (95% CI: 59.8–88.6; *p* < 0.0001). A significantly shorter time to result using BCID2 vs. conventional culture methods was already demonstrated in previous studies conducted in Northern America, Europe, South Africa and Australia [14,16,19,22,26,27,28]. Our study therefore confirms previous reports of the benefit of the BCID2 Panel for rapid microbial identification in BSI patients.

In our study, the 30-day mortality was significantly lower in BCID2 (17.3%) vs. pre-BCID2 (31.6%) patients (*p* = 0.019). Several studies investigated the impact of rapid microbial identification on patient mortality [3,10,11,25,27,30,36]. While some studies showed a significant reduction of mortality in association with rapid microbial identification [3,10,11,30,36], others did not [10,25,27]. A recent meta-analysis indicated that mortality is reduced only when rapid testing is accompanied with an antimicrobial stewardship program (ASP) [37]. Since this is the case at our participating hospitals, our observation that BCID2 implementation was associated with a significantly reduced mortality agrees with the reported meta-analysis by Timbrook et al. [37].

As expected, pathogens were identified in more patients following the implementation of BCID2 (98.8% vs. 87.9% patients; *p* = 0.003), and polydetections were more frequent post implementation of BCID2 (15.1% vs. 4.0% patients with two detected pathogens; *p* = 0.011). The frequency of polydetections upon implementation of BCID2 in our study is in line with the 10–20% polymicrobial bloodstream infections reported by Holma et al. [18].

The BCID2 Panel also enabled the identification of a high frequency of resistance genes (29.6% of detected pathogens). Of interest is the identification of the mecA/C methicillin resistance gene in 10/10 (100%) of detected *Staphylococcus epidermidis*. This highlights the benefit of the BCID2 Panel in identifying these clinically relevant pathogens, especially in the context of a reported high (up to 42%) prevalence of methicillin-resistant Staphylococcus aureus (MRSA) in Saudi Arabia [38,39]. Although the characterisation of resistance determinants is expected to impact the clinical decision for optimal targeted therapy, as observed in other studies [19,22,25,27,28], the duration of empirical antibiotics usage and the rate of antibiotics de-escalation were not statistically different between the pre-BCID2 and BCID2 study populations. Whether the absence of impact on antibiotics treatment adjustment observed in our study is due to the sample size, the study design (observational vs. interventional), or the already very effective standard of care (combined with an efficient ASP) remains to be investigated.

The concordance in pathogen identification between conventional methods and the BCID2 Panel in the BCID2 implementation phase was very good. Of the 98 detected pathogens, 78 (79.6%) showed a full or partial concordance, six (6.1%) were a mismatch, five (5.1%) were additional detections by BCID2 (*Candida auris*, *Staphylococcus epidermidis*, *Acinetobacter baumannii* complex, *Klebsiella pneumoniae*, *Streptococcus pneumoniae*), 5 (5.1%) were detection failures by BCID2 (not detected on-panel pathogens), and four (4.1%) were off-panel and only detected by conventional methods (*Corynebacterium* spp., *Kytococcus* spp., *Aeromonas sóbria*, *Enterococcus Avium Group D*). Of the five on-panel organisms not detected by BCID2, four were from polymicrobial specimens. The reason for frequent discordant organism identification in polymicrobial setting is unclear, but was previously reported in several studies [15,40,41,42,43]. The four off-panel organisms detected by conventional culture correspond to uncommon and usually non-pathogenic organisms, and are thus not expected to be clinically relevant. Therefore, the BCID2 Panel showed an overall reasonable performance in identifying clinically relevant pathogens, with few detection failures and a good concordance with conventional methods.

The strengths of this study include its multicentre design, its real-world setting, and the balanced study populations recruited over 6 months pre and post BCID2 implementation at the same three hospitals. This study presents some limitations, including the relatively low number of patients per group, the imbalanced number of patients enrolled per centre, which precluded a subgroup analysis per centre, the observational nature of the study, and the lack of documented data on the length of ICU stay. Further studies should include more patients from additional centres to confirm the present results. In addition, further molecular characterisation studies are needed to better understand the evolution of pathogens with antimicrobial resistance in the ICU setting.

## 5. Conclusions

The implementation of the BCID2 Panel at three UAE hospitals resulted in a significantly shorter time to result and a reduced 30-day mortality, compared to patients tested by conventional methods. This study therefore confirmed the work of others, supporting the use of the BCID2 Panel to improve clinical outcome in ICU patients with a bloodstream infection.

## Figures and Tables

**Table 1:** Patients’ characteristics.

	Pre-BCID2 Cohort (*n* = 99)	BCID2 Cohort (*n* = 86)	*p*-Value
Study population per centre, *N* (%)			<0.0001
Rashid Hospital	77 (77.8%)	37 (43.0%)	
Sheikh Khalifa General Hospital	13 (13.1%)	43 (50.0%)	
Mediclinic City Hospital	9 (9.1%)	6 (7.0%)	
Age in years, median (IQR)	56 (40–65)	65 (45–79)	0.001
Age class, *N* (%)			0.002
18–30 years old	8 (8.1%)	7 (8.1%)	
31–40 years old	17 (17.2%)	4 (4.7%)	
41–50 years old	13 (13.1%)	14 (16.3%)	
51–60 years old	26 (26.3%)	13 (15.1%)	
61–70 years old	19 (19.2%)	14 (16.3%)	
71–85 years old	16 (16.2%)	34 (39.5%)	
Sex, *N* (%)			0.433
Male	70 (70.7%)	56 (65.1%)	
Female	29 (29.3%)	30 (34.9%)	
Vital signs on ICU admission, median (IQR)			
Oxygen saturation (%)	98 (95.0–100.0) ^1^	97 (96.0–99.0) ^2^	0.194
Pulse rate (bpm)	93 (86.0–111.0) ^3^	101 (89.0–114.0)	0.177
Systolic blood pressure (mmHg)	107.5 (90.0–126.5) ^3^	108 (95.0–122.0)	0.695
Diastolic blood pressure (mmHg)	64 (52.0–74.25) ^3^	58.5 (49.0–68.0)	0.073
Comorbidities, *N* (%)			
Hypertension	46 (46.5%)	44 (51.2%)	0.557
Diabetes	45 (45.5%)	35 (40.7%)	0.554
Cardiac disease	29 (29.3%)	29 (33.7%)	0.530
Chronic lung disease	9 (9.1%)	28 (32.6%)	0.0001
Malignancy	15 (15.2%)	8 (9.3%)	0.269
Immunosuppressive treatment	6 (6.1%)	4 (4.7%)	0.753
SOT	3 (3.0%)	2 (2.3%)	1.000
HSCT	2 (2.0%)	0 (0.0%)	0.500
Number of comorbidities, *N* (%)			0.221
0	33 (33.3%)	20 (23.3%)	
1	17 (17.2%)	14 (16.3%)	
2	21 (21.2%)	29 (33.7%)	
3	20 (20.2%)	15 (17.4%)	
4 to 5	8 (8.1%)	8 (9.3%)	

^1^ *N* = 97 (two missing results). ^2^ *N* = 84 (two missing results). ^3^ *N* = 98 (one missing result). Abbreviations: bpm, beats per minute; HSCT, haematopoietic stem cell transplantation; IQR, interquartile range; mmHg, millimeters of mercury; SOT, solid-organ transplantation.

**Table 2:** Number of pathogens detected per positive blood culture in the pre-BCID2 and BCID2 study groups.

Study Population	Patients According to the Number of Detected Pathogens, *N* (%)		
	No Detections	One Detection	Two Detections
Pre-BCID2 phase (*n* = 99)	12 (12.1%)	83 (83.8%)	4 (4.0%)
BCID2 phase (*n* = 86)	1 (1.2%)	72 (83.7%)	13 (15.1%)

**Table 3:** Number of empirical antibiotics ordered in the pre-BCID2 and BCID2 study periods.

Study Population	Patients According to the Number of Ordered Empirical Antibiotics, *N* (%)				
	No Empirical Antibiotics	One Empirical Antibiotics	Two Empirical Antibiotics	Three Empirical Antibiotics	Unknown Number
Pre-BCID2 phase (*n* = 99)	1 (1.0%)	32 (32.3%)	42 (42.4%)	24 (24.7%)	0 (0.0%)
BCID2 phase (*n* = 86)	1 (1.2%)	37 (43.0%)	30 (34.9%)	17 (19.8%)	1 (1.2%) ^1^

^1^ Patient received empirical antibiotics, but the number and types of antibiotics were not recorded.

**Table 4:** Number of patients with antibiotics de-escalation (change from empirical to targeted antibiotics) in the pre-BCID2 and BCID2 study groups.

Study Population	Patients According to Antibiotics Change, *N* (%)		
	No Record of Antibiotics Change ^1^	Change to Targeted Antibiotics ^2^	Unknown ^3^
Pre-BCID2 phase (*n* = 99)	4 (4.0%)	94 (94.9%)	1 (1.0%)
BCID2 phase (*n* = 86)	3 (3.5%)	82 (95.3%)	1 (1.2%)

^1^ Patients with a record of ordered empirical antibiotics only; ^2^ Patients with a record of ordered empirical antibiotics and of antibiotics change to optimal therapy following test result (pathogen identification or susceptibility result); ^3^ Antibiotics change could not be determined due to a missing record on empirical and/or targeted therapy.

## Data Availability

The data presented in this study are available within the article or Supplementary Materials.

## Supplementary Materials

The following supporting information can be downloaded at https://www.mdpi.com/article/10.3390/diagnostics13142433/s1, Table S1: Number of detected organisms, resistance phenotypes (per conventional methods) and resistance genes (per BCID2 Panel) in patients of the pre-BCID2 (*n* = 87) and BCID2 (*n* = 85) implementation periods with at least one detected pathogen; Table S2: Resistance and susceptibility to antimicrobial treatment in the pre-BCID2 implementation phase; Table S3: Resistance and susceptibility to antimicrobial treatment in the BCID2 implementation phase; Table S4: Discordant pathogens detected by conventional culture vs. the BCID2 Panel among patients in the BCID2 implementation period (*n* = 24 discordant pathogens in *n* = 18 patients).
