# Supplementary material for: Clinical Impact of the BIOFIRE Blood Culture Identification 2 Panel in Adult Patients with Bloodstream Infection: A Multicentre Observational Study in the United Arab Emirates
Source: Diagnostics (Basel). 2023 Jul 21;13(14):2433. doi: 10.3390/diagnostics13142433 (PMC10378530; doi:10.3390/diagnostics13142433)
Supplement: Supplementary file 1 [file diagnostics-13-02433-s001.zip › diagnostics-2452703-supplementary.pdf]

Table S1. Number of detected organisms, resistance phenotypes (per conventional methods) and resistance genes (per BCID2 Panel) in patients of the pre-BCID2 (*n* = 87) and BCID2 (*n* = 85) implementation periods with at least one detected pathogen.

	**Pre-BCID2 organisms (*n* = 91)**		**BCID2-phase organisms (*n* = 98)**		
**Organism**	**N**	**Resistance phenotype**	**N**	**Resistance phenotype**	**Resistance genes (BCID2 panel)**
Klebsiella pneumoniae	19	ESBL (*n* = 3); AmpC (*n* = 1); CPE (*n* = 4); CRE (*n* = 1)	12	ESBL (*n* = 1); CPE (*n* = 1)	CTX-M (*n* = 4); OXA-48-like (*n* = 1); CTX-M + NDM (*n* = 1); CTX-M + OXA-48-like (*n* = 1)
Escherichia coli	14		13	ESBL (*n* = 1); AmpC (*n* = 1)	CTX-M (*n* = 7); *mcr-1* (*n* = 1); NDM (*n* = 1)
Staphylococcus epidermidis	10		10		*mecA*/*C* (*n* = 10)
Candida auris	0		13		
Candida tropicalis	2		6		
Staphylococcus aureus	6	MRSA (*n* = 2)	1		
Candida parapsilosis	4		5		
Staphylococcus capitis	5		4		
Staphylococcus haemolyticus	4		2		
Staphylococcus hominis	4		2		
Acinetobacter baumannii complex	1		4		
Enterococcus faecalis	2		2		
Enterobacter cloacae complex	2		2		
Serratia marcescens	1		2		
Enterococcus faecium	1		2		
Streptococcus pneumoniae	1		2		
Stenotrophomonas maltophilia	2		0		
Elizabethkingia meningoseptica	0		2		
Klebsiella aerogenes	0		2		OXA-48-like (*n* = 1)
Streptococcus viridans	0		2		
Corynebacterium spp.	1		1		
Candida albicans	1		1		
Candida krusei	1		1		
Pseudomonas aeruginosa	0		1		
Candida glabrata	1		0		
Enterococcus gallinarum	1		0		
Others	8 [1]		6 [2]		

[1] One each of the following: *Burkholderia cepacian, Klebsiella Ozaenae, Lactobacillus casei, Proteus mirabilis, Rothia mucilaginosa, Salmonella species, Weissella confusa, Leuconostoc pseudomesenteroide;* [2] One each of the following: *Staphylococcus* spp., *Bacteroides fragilis, Enterococcus Avium Group D, Kytococcus* spp., *Streptococcus oralis, Aeromonas sóbria.* Abbreviations: AmpC, class C beta-lactamase; CPE, carbapenemase-producing enterobacteriaceae; CRE, Carbapenem-resistant enterobacteriaceae; ESBL, positive for extended spectrum beta-lactamase; MRSA, methicillin-resistant *Staphylococcus aureus*.

Table S2. Resistance and susceptibility to antimicrobial treatment in the pre-BCID2 implementation phase.

Antimicrobial medication	Resistant	Susceptible	Total number of isolates tested	% tested isolates susceptible to medication
AMIKACIN	4	38	42	90.5
AMOXICILLIN/CLAVUL.	15	28	43	65.1
AMPHOTERICIN B	0	8	8	100.0
AMPICILLIN	31	12	43	27.9
CASPOFUNGIN	2	6	8	75.0
CEFACLOR	0	5	5	100.0
CEFAZOLIN	0	5	5	100.0
CEFEPIME	17	34	51	66.7
CEFIXIME	0	5	5	100.0
CEFOTAXIME	21	22	43	51.2
CEFTAZIDIME	20	29	49	59.2
CEFTAZIDIME AVIBACTAM	4	0	4	0.0
CEFTOLOZANE TAZOBACTAM	7	22	29	75.9
CEFTRIAXONE	16	28	44	63.6
CEFUROXIME	13	11	24	45.8
CIPROFLOXACIN	24	32	56	57.1
CLINDAMYCIN	9	12	21	57.1
COLISTIN	0	4	4	100.0
ERTAPENEM	7	34	41	82.9
FLUCONAZOLE	4	2	6	33.3
FLUCYTOSINE	1	7	8	87.5
FOSFOMYCIN	7	30	37	81.1
GENTAMICIN	12	52	64	81.3
IMIPENEM	8	34	42	81.0
LEVOFLOXACIN	11	14	25	56.0
LINEZOLID	0	27	27	100.0
MEROPENEM	7	39	46	84.8
MICAFUNGIN	1	7	8	87.5
MOXIFLOXACIN	6	6	12	50.0
NORFLOXACIN	13	23	36	63.9
PIPERACILLIN/TAZOBACTAM	11	36	47	76.6
RIFAMPICIN	2	15	17	88.2
TEICOPLANIN	0	15	15	100.0
TIGECYCLINE	0	20	20	100.0
TRIMETHROPRIM (TMP)	18	44	62	71.0
TOBRAMYCIN	6	18	24	75.0
VANCOMYCIN	4	24	28	85.7
VORICONAZOLE	1	8	9	88.9

Table S3. Resistance and susceptibility to antimicrobial treatment in the BCID2 implementation phase.

Antimicrobial medication	Resistant	Susceptible	Total number of isolates tested	% tested isolates susceptible to medication
AMIKACIN	3	28	31	90.3
AMOXICILLIN/CLAVUL.	17	13	30	43.3
AMPHOTERICIN B	0	12	12	100.0
AMPICILLIN	26	6	32	18.8
CASPOFUNGIN	0	9	9	100.0
CEFACLOR	4	4	8	50.0
CEFAZOLIN	4	1	5	20.0
CEFEPIME	16	19	35	54.3
CEFIXIME	4	3	7	42.9
CEFOTAXIME	19	12	31	38.7
CEFTAZIDIME	19	14	33	42.4
CEFTAZIDIME AVIBACTAM	0	8	8	100.0
CEFTOLOZANE TAZOBACTAM	3	10	13	76.9
CEFTRIAXONE	13	17	30	56.7
CEFUROXIME	18	7	25	28.0
CIPROFLOXACIN	16	21	37	56.8
CLINDAMYCIN	10	7	17	41.2
COLISTIN	0	5	5	100.0
ERTAPENEM	6	26	32	81.3
FLUCONAZOLE	2	8	10	80.0
FLUCYTOSINE	1	11	12	91.7
FOSFOMYCIN	6	20	26	76.9
GENTAMICIN	10	37	47	78.7
IMIPENEM	7	25	32	78.1
LEVOFLOXACIN	12	16	28	57.1
LINEZOLID	1	23	24	95.8
MEROPENEM	7	26	33	78.8
MICAFUNGIN	0	9	9	100.0
MOXIFLOXACIN	5	6	11	54.5
NORFLOXACIN	5	11	16	68.8
PIPERACILLIN/TAZOBACTAM	15	18	33	54.5
RIFAMPICIN	0	16	16	100.0
TEICOPLANIN	4	14	18	77.8
TIGECYCLINE	0	23	23	100.0
TRIMETHROPRIM (TMP)	12	39	51	76.5
TOBRAMYCIN	7	25	32	78.1
VANCOMYCIN	1	25	26	96.2
VORICONAZOLE	0	12	12	100.0

Table S4. Discordant pathogens detected by conventional culture *vs.* the BCID2 Panel among patients in the BCID2 implementation period (*n* = 24 discordant pathogens in *n* = 18 patients).

Patient	Organism identified by conventional culture	Organism identified by BCID2
**1**	*Staphylococcus hominis* [1]	*Staphylococcus epidermidis*
**2**	*Staphylococcus haemolyticus* [1]	*Staphylococcus epidermidis*
**3**	No detection	*Candida auris*
**3**	No detection	*Staphylococcus epidermidis*
**4**	No detection	*Acinetobacter baumannii* complex
**5**	No detection	*Klebsiella pneumoniae*
**6**	No detection	*Streptococcus pneumoniae*
**7**	*Corynebacterium* spp. [2]	No detection
**8**	*Pseudomonas aeruginosa*	No detection
**9**	*Kytococcus* spp. [2]	No detection
**10**	*Staphylococcus haemolyticus* [1]	No detection
**11**	*Aeromonas sóbria* [2]	No detection
**12**	*Enterococcus Avium Group D* [2]	No detection
**13**	*Candida krusei*	No detection
**14**	*Candida tropicalis*	No detection
**15**	*Serratia marcescens*	No detection
**16**	*Elizabethkingia meningoseptica* [2]	*Escherichia coli*
**17**	*Elizabethkingia meningoseptica* [2]	*Candida auris*
**18**	*Streptococcus viridans* [1]	*Staphylococcus* spp.

[1] Species not included in the BCID2 panel (but respective genus – *Staphylococcus* spp. and *Streptococcus* spp. – are on panel); [2] Off-panel microorganisms (not included in the BCID2 panel).
